# Anatomic All‐Inside Repair of the Anteroinferior and Posterosuperior Popliteomeniscal Fascicles for Hypermobile Lateral Meniscus

**DOI:** 10.1002/atn2.70150

**Published:** 2026-07-05

**Authors:** Seikai Toyooka, Yutoshi Osaki, Noriaki Arai, Hirotaka Kawano, Takumi Nakagawa

**Affiliations:** ^1^ Department of Orthopaedic Surgery Teikyo University School of Medicine Tokyo Japan

## Abstract

Hypermobile lateral meniscus refers to abnormal mobility of an otherwise morphologically normal meniscus without obvious tears, causing lateral knee pain due to catching or locking of the lateral meniscus during flexion. The most common cause is rupture or elongation of the popliteomeniscal fascicles (PMFs), which connect the lateral meniscus to the joint capsule around the popliteus tendon. Hypermobile lateral meniscus is most commonly managed with simple, minimally invasive all‐inside meniscal repair techniques or inside‐out suture techniques. However, nonanatomic suturing not only results in unphysiologic repair but may also compromise popliteus tendon function. We describe a simple all‐inside technique using a standard all‐inside meniscal fixation device to anatomically repair the anteroinferior and posterosuperior PMFs. This approach reproduces the native fascicular anatomy while avoiding perforation of the popliteus tendon. The technique restores lateral meniscal stability, minimizes the risk of iatrogenic popliteus tendon injury, and does not require additional posterior portals or graft harvesting, allowing it to be performed with commonly available devices.

## Graphical abstract

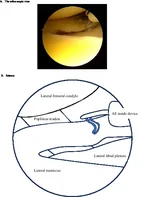

**VIDEO 1:** Description of the surgical technique: This video shows a surgical technique to repair popliteomeniscal fascicles anatomically. This approach reproduces the native fascicular anatomy while avoiding perforation of the popliteus tendon. The technique restores lateral meniscal stability, minimizes the risk of iatrogenic popliteus tendon injury, and does not require additional posterior portals or graft harvesting, allowing it to be performed with commonly available devices. Video content can be viewed at https://doi.org/10.1002/atn2.70150.

Hypermobile lateral meniscus is a pathological condition in which an otherwise morphologically normal lateral meniscus exhibits abnormal mobility, leading to lateral knee pain, catching, and locking.^1^, ^2^ Symptoms typically occur when the posterior part of the lateral meniscus translates excessively anteriorly or reduces abruptly during knee flexion.^3^, ^4^ This abnormal mobility is thought to result from rupture or elongation of the popliteomeniscal fascicles (PMFs), which connect the lateral meniscus to the joint capsule around the popliteus tendon.^2^, ^5^ The PMFs contribute to posterior stability of the lateral meniscus and play an important role in maintaining stability of the lateral compartment.^2^

A variety of arthroscopic techniques have been reported for the treatment of hypermobile lateral meniscus, including all‐inside and inside‐out meniscal repair, reconstruction of the PMFs using autologous grafts, and tightening of the posterior horn of the lateral meniscus with pull‐out sutures.^3^, ^4^, ^6^, ^7^, ^8^, ^9^ Among these, all‐inside repair using meniscal fixation devices is particularly attractive because it is simple, minimally invasive, and easily incorporated into routine arthroscopic practice. However, these techniques often focus on stabilizing the posterior part of the lateral meniscus without clearly targeting the anatomic origins and insertions of the anteroinferior and posterosuperior PMFs. The lateral meniscus is inherently more mobile than the medial meniscus and normally translates substantially with rollback of the lateral femoral condyle during deep knee flexion.^10^, ^11^ Therefore, overconstraint of the lateral meniscus is undesirable and may result in loss of motion, a sense of tightness, or persistent symptoms.

Nonanatomic suture placement may lead to unphysiologic repair and carries the risk of inadvertently penetrating the popliteus tendon, potentially causing pain, mechanical impingement, or repair failure.^12^, ^13^, ^14^ In this Technical Note, we describe a simple all‐inside technique that anatomically repairs the anteroinferior and posterosuperior PMFs using a commercially available all‐inside meniscal fixation device. By aligning the trajectory of the device with the anatomic course of each fascicle and carefully avoiding the popliteus tendon, this method allows anatomic restoration of PMFs function while preserving the advantages of a minimally invasive all‐inside approach.

## SURGICAL TECHNIQUE

### Surgical Indications

The primary indication for this technique is symptomatic hypermobile lateral meniscus. This condition is defined by the presence of lateral joint line pain and/or mechanical symptoms such as catching, locking, or a giving‐way sensation during knee flexion or activities like stair climbing, together with arthroscopic confirmation of excessive posterior translation or recurrent subluxation of the lateral meniscus. At arthroscopy, the meniscus is probed, and hypermobility is diagnosed when the posterior horn of the lateral meniscus translates beyond the apex of the lateral femoral condyle.

The technique is particularly suitable for patients in whom magnetic resonance imaging shows rupture or elongation of the anteroinferior and posterosuperior bundles of PMFs, as well as widening of the popliteal hiatus or popliteus tendon sulcus, suggesting that PMF insufficiency is the main cause of the abnormal mobility. However, it should be noted that magnetic resonance imaging does not always clearly show these lesions, and the diagnosis often relies primarily on arthroscopic findings.^5^

Relative contraindications include advanced degenerative changes in the lateral compartment, characterized by diffuse cartilage wear; irreparable lateral meniscal lesions, such as complex, degenerative, or markedly frayed tears; and a history of prior surgery around the popliteal hiatus that has resulted in substantial scarring, obscuring the PMFs anatomy and preventing reliable identification of the fascicles.

### Surgical Technique

The overall sequence of the procedure is shown in Video 1. The procedure is performed with the patient in the supine position under spinal or general anesthesia. A pneumatic tourniquet is applied to the proximal thigh and inflated at the discretion of the surgeon.

### Portal Placement and Diagnostic Arthroscopy

Standard infrapatellar anterolateral and anteromedial portals are established. The anterolateral portal is typically used as the viewing portal, and the anteromedial portal serves as the working portal. A systematic diagnostic arthroscopy is then performed to evaluate the patellofemoral joint, medial compartment, intercondylar notch, and lateral compartment. The lateral meniscus is probed to assess its morphology, the presence or absence of tears, and its mobility.

With the knee placed in valgus and flexion in figure‐of‐4 position, a probe is used to translate the posterior horn of the lateral meniscus anteriorly. Hypermobile lateral meniscus is diagnosed when the posterior horn readily rides over the apex of the lateral femoral condyle. In addition, hypermobility can be confirmed by performing an aspiration test, in which fluid is aspirated from the joint to observe abnormal excursion of the lateral meniscus.

With the knee in extension, the popliteus tendon and popliteus tendon sulcus are visualized from the lateral portal. The anteroinferior PMF is identified as a fascial band extending from the inferior margin of the lateral meniscus toward the posterior capsule along the popliteal hiatus. The posterosuperior PMF is visualized as a superior fascial structure arising from the posterior horn of the lateral meniscus and coursing toward the posterior capsule above the popliteus tendon. Disruption or elongation of these fascicles and widening of the popliteal hiatus or popliteus tendon sulcus are noted.

### All‐Inside Device and General Considerations

This technique uses a standard all‐inside meniscal repair device. In our practice, we employ a suture‐based device (FiberStitch; Arthrex, Naples, FL) that deploys 2 anchors connected by a nonabsorbable suture. Other similar all‐inside devices may be used according to surgeon preference and availability.

Throughout the procedure, the popliteus tendon is kept clearly in view, and care is taken to avoid its penetration by the device. The trajectory of the device should follow the native orientation of each PMFs, with 1 anchor placed at the meniscal attachment and the other at the capsular attachment. The all‐inside device is introduced through the anteromedial portal; insertion from the lateral portal is avoided because the trajectory is directed toward the popliteal vessels and nerve.

### Anatomic All‐Inside Repair of the Posterosuperior PMF

To repair the posterosuperior PMF, the knee is flexed further to approximately 100° to 110° and externally rotated as needed to improve visualization of the superior aspect of the popliteal hiatus. The arthroscope remains in the anterolateral portal, and the all‐inside device is again inserted through the anteromedial portal.

**FIGURE 1:**
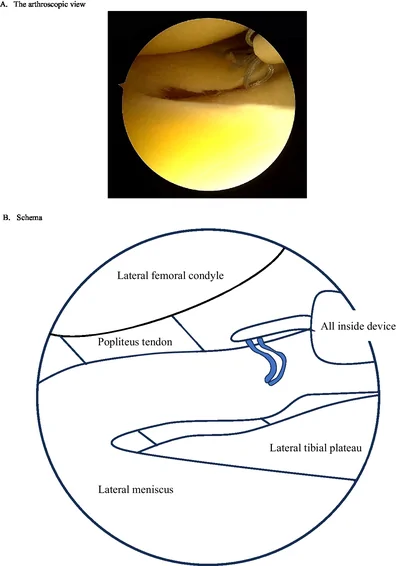
Front view of the lateral meniscus of the right knee. The origin of the posterosuperior popliteomeniscal fascicle (PMF) at the posterior horn of the lateral meniscus is identified above the popliteus tendon. At this location, the device needle is advanced into the meniscal tissue, taking care to avoid the articular surface and remain within the peripheral rim. The first anchor is deployed within the meniscus. The needle is then oriented toward the posterior capsule along the superior course of the PMF, maintaining a clearly superior trajectory to avoid penetrating the popliteus tendon. The second anchor is deployed in the posterior capsule, reproducing the anatomic insertion of the posterosuperior PMF. The suture is tensioned to restore the superior restraint while maintaining a physiologic range of meniscal motion.

The origin of the posterosuperior PMF at the posterior horn of the lateral meniscus is identified above the popliteus tendon. At this location, the device needle is advanced into the meniscal tissue, taking care to avoid the articular surface and remain within the peripheral rim. The first anchor is deployed within the meniscus. The needle is then oriented toward the posterior capsule along the superior course of the PMF, maintaining a clearly superior trajectory to avoid penetrating the popliteus tendon. The second anchor is deployed in the posterior capsule, reproducing the anatomic insertion of the posterosuperior PMF. The suture is tensioned to restore the superior restraint while maintaining a physiologic range of meniscal motion. Figure 1 shows this procedure.

### Anatomic All‐Inside Repair of the Anteroinferior PMF

**FIGURE 2:**
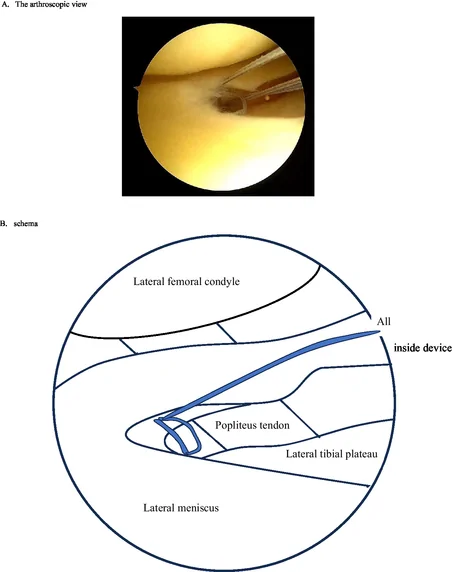
Front view of the lateral meniscus of the right knee. The all‐inside device is introduced through the anteromedial portal and positioned along the inferior margin of the posterior horn‐body junction of the lateral meniscus, corresponding to the origin of the anteroinferior popliteomeniscal fascicle (PMF). Under direct visualization, the device needle is advanced through the peripheral rim of the lateral meniscus at the anatomic origin of the anteroinferior PMF, and the first anchor is deployed within the meniscal substance. The needle is then redirected toward the posterior capsule along the expected course of the anteroinferior PMF, taking care to remain inferior to the popliteus tendon and outside its substance. The second anchor is deployed in the capsule at the anatomic insertion site of the anteroinferior PMF.

The all‐inside device is introduced through the anteromedial portal and positioned along the inferior margin of the posterior horn‐body junction of the lateral meniscus, corresponding to the origin of the anteroinferior PMF. After confirming the location of the popliteus tendon, the device needle is advanced through the peripheral rim of the lateral meniscus at the anatomic origin of the anteroinferior PMF, and the first anchor is deployed within the meniscal substance. To secure as much meniscal fibers as possible with a vertical mattress suture, the first anchor needle should be directed upward. The needle is then redirected toward the posterior capsule along the expected course of the anteroinferior PMF, taking care to remain inferior to the popliteus tendon and outside its substance. The second anchor is deployed in the capsule at the anatomic insertion site of the anteroinferior PMF. The suture is tensioned to draw the peripheral meniscus toward the capsule while avoiding overconstraint, thereby reconstructing the fascicle and reproducing the native orientation of the anteroinferior PMF. Depending on the degree of hypermobility and tissue quality, additional sutures may be placed along the anteroinferior or posterosuperior fascicles using the same principles. Care is taken to avoid overtightening, which may restrict lateral meniscal excursion and lead to overconstraint of the lateral compartment. Figure 2 shows this procedure.

### Final Assessment

After suture fixation, the stability of the lateral meniscus is reassessed with a probe. Posterior translation and subluxation should be markedly reduced compared with the prerepair condition. The knee is then taken through a full range of motion while visualizing the popliteal hiatus to confirm that the popliteus tendon sulcus has been effectively closed and that the tendon glides smoothly without impingement.

### Postoperative Rehabilitation

Postoperative rehabilitation is individualized according to patient factors and concomitant procedures; however, a typical protocol is as follows. Full weight‐bearing in extension with the use of crutches is permitted immediately after surgery. A knee extension brace is applied for the first 2 weeks to protect the repair and restrict range of motion. Controlled passive and active‐assisted range‐of‐motion exercises are initiated after 2 weeks, with gradual progression as tolerated. Deep flexion beyond 90° in a weight‐bearing position is avoided for the first 3 months to minimize stress on the repaired PMFs.

Quadriceps and hamstring strengthening is started early with isometric exercises and is then advanced to closed‐chain exercises as tolerated. Running is generally allowed at approximately 3 months postoperatively, whereas return to pivoting or cutting sports is typically permitted between 4 and 6 months, provided that muscle strength, neuromuscular control, and symptoms are acceptable.

## DISCUSSION

Hypermobile lateral meniscus is often underdiagnosed because the lateral meniscus frequently appears morphologically normal on preoperative imaging.^5^ Accurate diagnosis requires recognition of excessive posterior translation on probing and careful assessment of the popliteal hiatus and the PMFs. Rupture or elongation of the anteroinferior and posterosuperior PMFs is considered a major pathoanatomic factor leading to instability of the lateral meniscus.^2^, ^15^

Conventional all‐inside repair techniques for hypermobile lateral meniscus are widely used because they are simple and can be performed through standard anterior portals. However, when the specific target of repair is not clearly defined, sutures may be placed in a nonanatomic fashion and fail to restore the native function of the PMFs. In addition, unintended trajectories of all‐inside devices may penetrate the popliteus tendon, resulting in pain, restriction of motion, or the need for revision surgery.

The technique described in this report emphasizes anatomic restoration of the PMFs by intentionally aligning the all‐inside repair device with the anteroinferior and posterosuperior bundles. By identifying the meniscal origin and capsular insertion of each PMFs and reproducing their orientation using suture‐based anchors, the native restraining function of the PMFs can be restored without graft harvest or the creation of additional posterior portals. The use of a commonly available device such as FiberStitch allows a low‐profile construct with broad applicability while maintaining the minimally invasive nature of all‐inside repair.

An important advantage of this technique is the potential reduction in the risk of iatrogenic popliteus tendon injury. Because the popliteus tendon is visualized throughout the procedure and the trajectory of the device is planned with reference to the tendon, penetration of tendon fibers during anchor deployment can be avoided. The anteroinferior bundle is reconstructed inferior to the tendon and the posterosuperior bundle is reconstructed superior to the tendon, thereby respecting the natural anatomy of the popliteal hiatus.

This technique has several limitations. The advantages, disadvantages, pearls, and pitfalls of this procedure are shown in Tables 1 and 2. First, it requires detailed familiarity with the arthroscopic anatomy of the popliteal hiatus and PMFs, which may not be routinely appreciated by all surgeons. Second, long‐term clinical outcomes following this specific anatomic all‐inside PMF repair technique remain to be determined. Third, the procedure may be technically demanding in knees with substantial scarring, synovitis, or a history of lateral compartment surgery that obscures the PMFs. Finally, although the method is shown using a specific all‐inside device, surgeons must adapt the underlying principles to the characteristics and constraints of the devices available in their own practice. Despite these limitations, anatomic all‐inside repair of the PMFs offers a logical and reproducible strategy for addressing the fundamental pathology of hypermobile lateral meniscus. By combining detailed arthroscopic visualization with targeted suture placement, this technique provides a practical option for restoring lateral meniscal stability while minimizing the risk of popliteus tendon injury.

**TABLE 1:** Advantages and Disadvantages of Anatomic All‐Inside Repair of the Anteroinferior and Posterosuperior Popliteomeniscal Fascicles

Advantages	Disadvantages
• Anatomic restoration of both the anteroinferior and posterosuperior PMFs directly addresses the primary pathoanatomy of hypermobile lateral meniscus. • The technique is entirely arthroscopic and all‐inside, requiring only standard anterior portals and no graft harvest or accessory posterior portals. • Continuous visualization and deliberate avoidance of the popliteus tendon reduce the risk of iatrogenic tendon injury. • The method can be adapted to different all‐inside devices by applying the same anatomic and technical principles.	• Long‐term clinical outcomes and durability of this specific anatomic all‐inside PMF repair technique remain to be established.

PMF, popliteomeniscal fascicle.

**TABLE 2:** Pearls and Pitfalls of Anatomic All‐Inside Repair of the Anteroinferior and Posterosuperior Popliteomeniscal Fascicles

Pearls	Pitfalls
• Careful probing with the knee in flexion and valgus is essential to reproduce the patient's symptoms and confirm hypermobility of the lateral meniscus. • Visualization of the popliteal hiatus is greatly improved by viewing from the lateral portal with the knee in extension. • The popliteus tendon should be clearly identified and kept within the visual field throughout device deployment, and used as a landmark: the anteroinferior PMF is reconstructed inferior to the tendon, and the posterosuperior PMF superior to it. • The all‐inside device is introduced through the anteromedial portal to avoid directing the needle toward the popliteal neurovascular bundle, and its trajectory is planned to follow the native course of each PMFs. • Sutures are tensioned gradually, with repeated probing of the lateral meniscus to confirm restoration of stability without overconstraint.	• Inadequate visualization of the popliteal hiatus and PMFs increases the risk of nonanatomic suture placement and persistence of hypermobility. • Insertion of the all‐inside device from the lateral portal may direct the needle toward the popliteal neurovascular bundle and should therefore be avoided. • Penetration of the popliteus tendon by the device can result in pain, restriction of motion, and the potential need for revision surgery. • Excessive tightening of the sutures or placement of too many anchors may overconstrain the lateral meniscus, restrict its physiologic excursion, and lead to residual lateral joint line pain or a sense of tightness, whereas capturing too little meniscal tissue may result in insufficient tension and an increased risk of recurrence.

PMF, popliteomeniscal fascicle.

## DISCLOSURES

The authors (S.T., Y.O., N.A., H.K., T.N.) declare that they have no known competing financial interests or personal relationships that could have appeared to influence the work reported in this article.
